# Sustainable Applications for the Valorization of Cereal Processing By-Products

**DOI:** 10.3390/foods11020241

**Published:** 2022-01-17

**Authors:** Charis M. Galanakis

**Affiliations:** 1 Research & Innovation Department, Galanakis Laboratories, 73131 Chania, Greece; cgalanakis@chemlab.gr; 2 Department of Biology, College of Science, Taif University, Taif 26571, Saudi Arabia; 3 Food Waste Recovery Group, ISEKI Food Association, 1190 Vienna, Austria

**Keywords:** brewers’ spent grain, oat, arabinoxylans, proteins, biorefinery, ultrafiltration, bakery products

## Abstract

This review article revises the sustainable practices and applications to valorize valuable components recovered from cereal processing by-products. After introducing cereal processing by-products, their healthy compounds, and corresponding functional properties, the article explores reutilization opportunities of by-products emphasizing specific sources (e.g., oat and wheat bran, distillers’ dried grains, etc.) and the biorefinery approach. Proteins and soluble dietary fibers such as arabinoxylans are of particular interest due to their content in the cereal processing by-products and their easy extraction based on conventional technologies such as enzyme-assisted extraction and membrane filtration. Non-thermal technologies have also been suggested to improve sustainability recovery approaches. Finally, the article discusses the different applications for the recovered high-added value compounds that span across biotechnology, foods, and bakery products.

## 1. Introduction

Cereals comprise an essential source in the human diet and a significant part of livestock feed for thousands of years, while their processing represents a substantial asset to the food production chain [1]. Epidemiological studies have indicated that the consumption of whole cereal grains is correlated with a lower risk of developing cardiovascular and chronic diseases such as cancers, diabetes, and obesity [2]. The health benefits of cereal grains have been attributed to their content in high amounts of lipids, proteins, dietary fiber, tocopherols, Vitamins B, and E [3]. On the other hand, these compounds are concentrated in the hull, bran, and cereal germ that are removed from foods during cereal manufacturing.

Cereal manufacturing includes different processes such as dry and wet milling, malting, and pearling, which produce by-products of varying nature and chemical compositions, e.g., wheat and maize bran, rice bran and corn germ oil, and distiller’s dried grains [4]. Nevertheless, all of them contain valuable nutritional components (similar to whole grains) that could be converted to biofuels, bioplastics and biopolymers. Alternatively, they could be recaptured and reused in the food chain, finding innovative nutraceutical and pharmaceutical applications [5] as well as applications in fermentative for the production of bioactive microbial metabolites, enzymes, single cell proteins and oils [6]. For example, alkylresorcinols (existing only in rye and wheat bran) could reduce cholesterol absorption, and reduce the risks of chronic diseases such as obesity and diabetes [7]. Besides, the market of functional food ingredients supporting the immune system will grow in the post-pandemic era [8,9]. At the same time, there is an urgent need to valorize food processing by-products in order to improve food security in the next decades [10]. Cereal processing by-products are rich in β-glucan that has been proposed as a bioactive food compound against SARS-CoV-2 infection [11].

Besides, the current managing practices that discharge cereal processing by-products to the environment are not sustainable. This article revises the valorization opportunities of cereal processing by-products giving emphasis on target compounds and highlighting their most important characteristics as well as corresponding sustainable applications in different sectors.

## 2. Cereal Processing By-Products and Target Compounds

Table 1 shows the different processes during the dry and wet milling of varying cereal grains (corn, rice, and wheat). Dry milling is one of the oldest practices of cereal manufacturing, providing cereal flours and grains. It includes cleaning, magnetic, sieve, and disc separators, to remove impurities, an aspiration to remove dust, and other procedures such as conditioning (moistening of kernels). In wet milling, soaked grains are ground before separating particular components such as oil, dietary fiber, starch, and proteins [12]. The main generated by-products of these processes include hulls, germ, bran, and wastewater from polishing. The ratio yields of significant products and derivatives are affected by the milling degree and the cultivar [5]. For instance, in paddy rice processing, white rice (endosperm) represents almost 70% of the whole grain’s weight, while by-products such as husk, bran, and germ represent 20, 10, and 2%, respectively [13].

Table 2 summarizes the functional components of cereal processing by-products. Rice bran high amounts of carbohydrates (~50%), oil (~20%), proteins (~13%), and dietary fiber (~11%) that is comprised of β-glucan, pectin, and gum. It also contains significant amounts of minerals such as magnesium, phosphorous, and iron, as well as bioactive phytochemicals such as ferulic acid, phytic acid, squalene, polycosanols, phytosterols, oryzanols, and tocotrienols [14,15]. On the other hand, corn bran is mainly composed of insoluble dietary fibers such as hemicelluloses (ca. 700 g/kg), cellulose (ca. 240 g/kg), and lignin fractions (ca. 10 g/kg) [16]. Compared to other cereals bran, corn bran has the highest dietary fiber content, tocopherols, and polyphenolic compounds that have well-known antioxidant properties and can be used in different applications. For example, they can be used as bioactive compounds in cosmetics or natural substitutes (antioxidant preservatives, stabilizers, emulsifiers, and coloring agents) in foods preventing the potential adverse effects associated with the consumption of the artificial ingredients [17,18,19,20]. Corn bran is a rich source of ferulic acid compared to other vegetables, fruits, and cereals, while sorghum bran contains high amounts of 3-deoxyanthocyanidins. The latest is a rare class of flavonoids with intense cytotoxic activities. Besides, the oil recovered from rice bran is rich in tocopherols, phytosterols, and tocotrienols and particularly γ-oryzanols, which show ten-fold higher antioxidant properties compared to tocopherols [20,21,22]. 

The processing by-products of oat comprise the most typical examples of underestimated substrates with high valorization potential. Oats are rich in proteins (11–20 g/100 g) and β-glucan (2.2–7.8 g/100 g) [28,29], which have been correlated with the control of normal cholesterol’s level in the blood [30]. This property of β-glucan and proteins as well as their viscoelastic characteristics allow considering them as ideal additives in foods and confectionary [31,32,33]. For instance, hydrolysis of oat’s carbohydrates (e.g., starch, maltose, and β-glucan) has been implemented for the industrial development of nondairy formulations such as milk alternatives, cream, ice cream, and other products consumed by lactose-intolerant individuals [34,35,36]. The biodegradation process is conducted using multi enzymatic cultures, and the viscoelastic properties of the hydrolysates are optimized using kinetics modeling [37]. The by-product of this process (oat mill waste) is typically utilized as livestock feed, although it is still rich in β-glucan and proteins. Thus it could be used in different food applications, e.g., to mimic and replace fat of cheese and yogurt [38,39,40]. Sibakov et al. [41] applied a dry fractionation to remove lipids from oat grain and extract β-glucan from oat grains. This recovery approach produced another by-product rich in unfolded globulin that aggregates due to the acidic conditions [42,43]. Aggregated proteins show poor functionality, e.g., decreased solubility, viscosity, emulsification, and foaming ability [44,45]. On the other hand, the aggregation of proteins could be utilized for the structure formation of lactic acid fermented products. Thereby, it is essential not only to recover target compounds, but also to develop tailor-made applications [46].

Wheat bran is rich in dietary fiber and particularly arabinoxylans, which are non-starch polysaccharides with high ratios of arabinose/xylose β-glucans, fructans, cellulose, and lignin [25,47]. Arabinoxylans can be found in the cell walls of all cereal grains interlinked with ferulic and other phenolic acids [47]. They present different structures and properties, namely water retention, viscoelastic properties, high nutritional characteristics, and health-promoting impacts, e.g., reduce the glucose levels in the blood [18]. Wheat bran’s fiber contributes to several beneficial physiological effects (e.g., laxation, acceleration of intestinal transit, increase in fecal bulk, and blood cholesterol attenuation) [5,48]. More specifically, arabinoxylans can lower blood sugar levels and are widely used in food (e.g., as sweetener, medicine, and cosmetic industries) [49,50]. On the other hand, phenolic acids show high oxidation resistance and exert in vitro anti-inflammatory and anticancer effects [51].

Rye bran is also rich in dietary fiber-containing higher contents of β-glucan and fructan. Still, lower amounts of arabinoxylans than wheat (5%, 7%, and 23% compared to 2.5%, 3%, and 26%, respectively) [52]. Arabinoxylans have prebiotic properties (e.g., bifidogenic enhancement) and can be used as fat replacers and functional components in meat, dairy and bakery products [18,38,53,54]. The recovery of arabinoxylans from wheat, rye, and other cereals bran is typically conducted using several enzymatic, chemical, and hydrothermal treatments that lead to arabino-oligosaccharides with different lengths. Subsequently, the challenge during extraction is to match the structure and size of the recovered arabinoxylans with the required properties of the final products. For instance, the application of alkali or acid solutions causes the breakage of ferulic acid, leading to the extraction of oligosaccharides with a low arabinose substitution ratio [18]. 

Barley is the primary raw material for the production of beer. During malting and brewing, the main leftovers of the process (~85%) are the well-known brewers’ spent grains (BSG). BSG contains high amounts of lignin (28%) and non-cellulosic polysaccharides (28%), including β-glucan and arabinoxylans, and cellulose (17%), as well as has considerable amounts of proteins and polyphenols [18]. BSG is merchandized as livestock feed, although it comprises an excellent source for recovering their food-grade components. The proteins of barley, wheat, corn, and rice processing by-products have better functional properties compared to those of the endosperm due to their content in essential amino acids and their valorization potential in the market of extruded and cereal-based baked products is high [18,55]. The fermentation of BSG with different lactic acid bacteria (e.g., with Leuconostoc pseudomesenteroides DSM20193 and Weissella confusa A16 as suggested by Koirola et al. [56]) has been examined in different applications such as yoghurt [57], pasta [58] and bread [59].

## 3. Recovery Approaches 

Table 3 presents different approaches for the recovery of valuable compounds from different cereal processing by-products. As shown above, cereal bran, germ, and husk are rich in functional macromolecules such as proteins, β-glucan, arabinoxylans, pectin, and smaller molecules such as ferulic acid and vitamins. At first, each of these by-products should be pre-treated using grinding, wet milling, or drying [46]. The second step requires the separation of macro-and micro-molecules using different techniques such as isoelectric solubilization or alcohol precipitation. Isoelectric solubilization/precipitation can separate proteins more selectively (due to their charge) compared to alcohol’s addition that precipitates proteins together with soluble (β-glucan, arabinoxylans, and pectin) and insoluble (e.g., lignin and cellulose) dietary fiber [46]. Another approach for the separation of macro- and macro-molecules in cereal processing by-products is the utilization of membrane processes. For example, Patsioura et al. [32] used a simple ultrafiltration step and a 100 kDa-polysulfone membrane to concentrate β-glucan from oat processing waste in a cross-flow module and remove smaller molecules in the permeate. The proposed process operated at low transmembrane pressure (≤2 bar), but it was not selective enough to separate β-glucan from proteins. 

**Figure 1:**
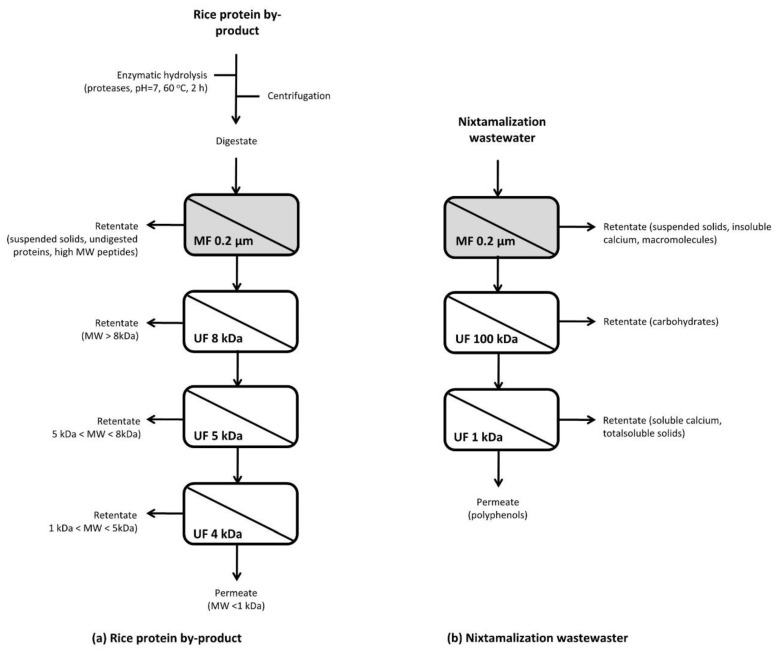
Process flow for the recovery of valuable compounds from (**a**) rice protein by-product and (**b**) nixtamalization wastewater using integrated membrane processes. MF: microfiltration, UF: ultrafiltration, MW: Molecular weight. Remade from [16,30,65].

In order to increase selectivity, many researchers suggested the application of sequential ultrafiltration systems for the valorization of by-products derived from different cereal processing by-products (e.g., from corn, rice, and wheat). The advantage of combined membrane systems is the synergy resulting from their integration, with overall significant benefits in plant compactness, product quality, energy efficiency, and environmental impact [18]. For instance, Ferri et al. [60] used a combined treatment with proteases (Protamex, alcalase, and neutrase) and sequential membrane processes for this purpose (Figure 1a). Rice by-products were first subjected to enzymatic hydrolysis (ph = 7 and 60 °C for 2 h) before recovering the digestate with centrifugation and its sequential fractionation with one microfiltration (0.2 μm) and three polyethersulfone ultrafiltration (8, 5, and 4 kDa, respectively) steps. The retentate with the highest peptide content was the one recovered with the 8 kDa membrane. Compared to polysulfone, the polyethersulfone membrane is less hydrophobic, allowing the rapid absorption of polar compounds (e.g., polyphenols) and the release of soluble dietary fiber in the permeate [40]. In another effort, Castro-Muñoz and Yáñez-Fernández [65] used a combination of a hollow fiber microfiltration (0.2 μm) and an ultrafiltration (100 kDa, UFP-100-E-4A) membrane (Figure 1b). The first retentate was rich in suspended grain particles and macromolecules, allowing its application as a carbon source to generate biogas, bioethanol, and relevant biotechnological products. The second retentate contained carbohydrates that could be further processed with a 1 kDa ultrafiltration membrane for the recovery of soluble calcium that can be reused in the maize’s nixtamalization process and a polyphenol-rich permeate for food, pharmaceutical, and cosmetic applications.

The extraction of proteins from precipitates or concentrates are typically conducted using the alkaline solution (e.g., 0.1 M NaOH at 60 °C for 60 min for proteins extraction from BSG) and subsequent filtering (180 µm) of the supernatant, and acid precipitation with 2.0 M citric acid addition (pH modification to 4.0) [66], or by adding Na_2_HPO_4_ in sodium dodecyl sulfate solution [67]. For example, Idris et al. [61] investigated the recovery of proteins from wheat bran. At first, the adherent endosperm was removed from wheat bran with brushing before being extracted using the alkaline treatment and isoelectric precipitation. This approach led to high nitrogen dispensability with minimum and maximum protein solubility pH values of 5.5 and 11.5, respectively. 

Alkaline extraction has also be used for the recovery and fraction of arabinoxylans from cereal processing by-products since under these conditions, and the hydroxyl ions accelerate cellulose’s swelling and intermolecular hydrogen bonds’ disruption [68]. Alkaline also hydrolyses the carboxyl and acetyl groups of benzyl groups and uronic acids of lignin. The ester linkages between arabinose residues and the ferulic acid, leading to the solubilization of hemicellulose from cell insoluble matrix. For example, Mandalari et al. [63] reported that the sequential fractionation of BSG and wheat bran using KOH of increasing strength (0.5, 1.0, and 4.0 M) of growing strength and 50 mM Na_2_CO_3_ allowed the extraction of arabinoxylans with a lower ratio of arabinose/xylose as a factor of alkali’s power. 

However, there are concerns about the detrimental effects of this technique on proteins’ and arabinoxylans’ functionality and nutritional value, restricting their applications as additives in food products [18]. For example, the protein extracted using alkaline is poorly soluble, while the increased alkali strength results in the recovery of arabinoxylans with lower molecular [69]. The foaming and emulsification properties and the solubility of the isolated proteins could be increased using enzymes, e.g., specific proteases that modify proteins smoothly without destroying any amino acids [70]. This process generates a mixture of peptides owing different molecular weights and lengths as well as physicochemical and functional properties compared to non-hydrolyzed proteins, e.g., protein hydrolysates extracted from BSG exhibit immunomodulatory properties that could help the body against inflammatory diseases [71]. In order to increase proteins’ extractability, relevant biotechnological approaches have been suggesting the co-generation of other products, and the low yields of target peptides have limited their application [18]. 

## 4. Emerging Technologies

Eco-friendly (e.g., non-thermal) technologies [45,72,73,74,75,76,77] could be used for the transformation of by-products into innovative products, increasing profit and securing the sustainable development of the food industry. These techniques (e.g., ohmic heating, high-pressure processing, pulsed electric field, microwaves, high-intensity ultrasounds, and others) have been successfully applied in different substrates (e.g., to treat meat, eggs, seafood and surimi, tomatoes, soy albumin, carrots, whey, and broccoli). In particular, they showed promising results in terms of minimum proteins’ degradation, improvement of proteins’ gelling capacity, apparent digestibility, emulsifying capacity, and foaming capacity [45,78,79,80]. 

For instance, Tang et al. [62] applied ultrasounds to recover proteins from barley spent grain, referring to a yield of 104 mg/g under the optimum conditions (solid-to-liquid ratio of 2 g/100 mL and intensity of 88.2 W/100 mL of extractant). This yield (145.6 mg/g) can be further enhanced by coupling ultrasonic-assisted extraction with ultrafiltration using a 30-kDa membrane. Phongthai et al. [81] investigated the extraction of proteins from rice bran, referring to a yield of ~4.7% under the optimum conditions of 0.99 g/10 mL solid-to-liquid ratio, 76% sonication amplitude, and 18 min extraction time. Moreover, the proteins were hydrolyzed using three enzymatic preparations (Subtilisin A, Actinase E, and Neutrase 0.8 L), resulting in different hydrolysis degrees (e.g., 20%, 14%, and 6%, respectively). By increasing the hydrolysis degree, the foam capacity decreased, and the antioxidant capacity increased, resulting in peptides with different properties and potential applications. In another study, Phongthai et al. [64] used microwaves to extract proteins from rice bran. The optimum conditions were found to be a power of 1000 W, a water solid-to-liquid ratio of 0.89 g bran/10 mL, and an extraction time of 90 s. Microwaves are enhanced by 1.5 fold the protein yield of alkaline extraction. By applying a lower alcalase hydrolysis degree of 5%, the emulsification and foaming capacities increased, and the antioxidant capacity decreased.

## 5. The Biorefinery Concept

The modern bioeconomy requires not only the application of sustainable technologies, but also their integration in the biorefinery concept for the conversion of biomass to a range of biobased products for food, energy, textile, and other industrial applications [82]. Biobased products could replace products manufactured from fossil fuel feedstocks taking into account that renewable biomass prices have slowly and steadily decreased. Over the last years, the utilization of commonly used biomass materials (e.g., potato, wheat, sugar beet, and corn) as a source of glucose for the production of biofuels and biobased chemicals has been partially commercialized [18]. Besides, the feasibility of bioethanol production and appropriate processing steps from corn has been demonstrated by performing calculations that scale-up benchtop or pilot plan operations [83].

The first generation of bioethanol production is based on transforming the whole grain kernel by grinding and mixing with enzymes and water to progress the degradation of starch [84]. At this process, kernel’s components (e.g., proteins, dietary fiber, and germ) are not fermented, but are concentrated into a by-product, the so-called distillers’ dried grains with solubles (DDGS). It is estimated that 100 kg of grains can generate ~40 L of ethanol, ~32 kg of DDGS, and ~3 kg of CO_2_ [85]. Another sector contributing to the increasing amounts of DDGS is whiskey’s distilleries that produce potable ethanol using blended grains of barley, rye, wheat, and maize. This substrate is more attractive compared to the DDGS derived from distilleries, as it possesses a food-grade nature. Thus, the corresponding recovered products could be used directly in food formulations [86].

DDGS is a heterogeneous material whose rich composition in protein, lipids, carbohydrates, and other valuable ingredients varies depending on the initially blended cereals [87,88]. DDGS is mainly used as livestock feed, but its composition allows considering it as a substrate that can generate numerous high added-value biobased products within the biorefinery concept [18]. To this line, two approaches could be used. In the first case, the grains are subjected to dry milling or the quick germ technique that starts by soaking the whole grain in water up to 3 h at 60 °C. Thereafter, components such as germ meal, oil, and arabinoxylans are recovered from the residual grains before the starchy endosperm fermentation, saccharification, and bioethanol production [89,90]. In the second case, the DDGS and its intermediate products are directly fractionated into valuable components such as xylooligosaccharides, protein, phenolic acids, oils, and phytosterols [91]. The selected process should not be affected by the variability and the composition of the feedstock and should be easily incorporated into existing production processes. From an economic perspective, the thermal processing of intermediate products is the most expensive process of bioethanol production [83].

The valorization of wheat bran also fits well with the biorefinery concept. Wheat bran includes approximately 15% of wheat kernel constituents and is rich in non-starch carbohydrates (55–60%, dry matter based), starch (14–25%), and protein (13–18%). Other minor constituents include fat (3–4%), minerals (3–8%), and other components like lignans, flavonoids, phenolic compounds, polyols, amino acids, and organic acids [92,93]. The non-starch carbohydrates of the wheat bran are mainly composed of soluble dietary fiber and particularly arabinoxylan (52–70%) [48]. In order to convert wheat bran to biobased products, several pretreatment methods are required such as acid hydrolysis, solubilization of lignin and hemicelluloses with organic solvents, enzymatic depolymerization of arabinoxylans, wet alkaline oxidation, and steam explosion, among others [94,95]. After the hydrolysis of cellulose and hemicelluloses, the released sugars are converted to bioethanol, glycerol, butanol, organic acids (e.g., levulinic, acetic, and formic and), and other products through submerged fermentation [96]. The products can be further transformed into other biobased products, e.g., polyethylene (used in packaging) by dehydrating ethanol to ethylene, polymerizing it, or polybutylene succinate produced by the esterification of succinic acid and butane-1,4-diol [95].

## 6. Applications

As shown above, primary and secondary by-products of cereals processing contain several high added-value compounds that could be converted into different commodities for different sectors. Besides, numerous applications have today been commercialized, e.g., Promitor Soluble Fiber from Tate & Lyle, Stabilized Rice Bran from NutraBio, Gama Oryzanol from Swanson Health Products, Oat Fiber Plus Tablets from Now Foods, and Life Extension NK Cell Activator from Swanson Health Products [18]. 

Cereal processing by-products are rich in nutrients that could replace the typical carbon sources in media preparations used in industrial enzyme production and microbial processes [97]. For instance, they could be utilized for the production of microbial enzymes (e.g., xylanase, cellulases, proteases, and amylases) and single-cell protein at low cost and high production yields [98,99,100,101]. The industrial production of enzymes is based on the cultivation of certain bacterial and fungal strains (mainly *Aspergillus oryzea*, *Aspergillus niger*, and *Bacillus subtilis*) that produce them during their metabolism. For instance, Gomathi et al. [102] investigated carboxymethyl cellulose production by *Aspergillus flavus* using submerged fermentation and wheat bran as a substrate. Hashemi et al. [103] studied the production of α-amylase using *Bacillus* sp. KR-8104 as a starting culture in a submerged fermentation system. The addition of BSG (by 5%, *w*/*v*) and removing dextrin from the culture medium enhanced α-amylase production by five times, making it more economically sustainable. Solid-state fermentation using different agricultural by-products (e.g., rice husk, gram bran, maize bran, wheat bran, and straw) has also been tested to produce xylanase by *Bacillus subtilis* ASH. Among the different substrates, wheat bran provided the highest xylanase yields because it contained high amounts of carbohydrates and proteins [104,105]. In a similar effort, Tanasković et al. [106] proposed the utilization of Bacillus sp. TMF–2 for the solid-state fermentation of wheat bread, as this strain triplicated the soluble phenolic content of wheat bran, accelerating a significant increase of antioxidant capacity and free radical scavenging activity.

The secondary cereal processing by-products (e.g., BSG and DDGS) have been investigated in different food applications, e.g., to fortify bakery products, snacks, cookies, and flavors. To this line, several pretreatment methodologies (e.g., chemical, biochemical, hydrothermal, enzymatic, or physical like pre-soaking, milling, and extrusion) have been applied for the retardation of rancidity and the optimization of end-products’ quality. Moreover, safety assays should be conducted together with studies dealing with the rheological, functional, and sensory properties of the developed food products before their usage in commercialized commodities. 

The most popular application of cereals’ bran is their usage in baked products in order to increase their content in dietary fiber. The percentage of cereals’ bran in bread may vary according to the application and the needed properties, e.g., how much we want to increase bread’s fiber content, how much we want to decrease its glycemic index, and what kind of sensory properties are needed. The addition of bran in baked products may affect the quality negatively and sensory characteristics of baked products, e.g., causing porosity and elasticity changes, increasing bitterness due to the contained phenolic compounds (pinoresinol and syringic acid), reducing bread’s volume, or reducing nutrients absorption due to their increased content in phytic acid [18,107,108]. Likewise, cereals’ bran contains high amounts of oil that are susceptible to oxidation, causing off-flavors and in the baked products. Therefore, oil’s removal from bran is necessary before application. 

For example, the incorporation of wheat bran in a high percentage of 15–20% has been referred to as increased water immobilization during dough making and decreases its crumb textural quality and volume [109]. Coda et al. [110] referred that a 160 μm-particle size of wheat bran resulted in high volume bread. Le Bleis et al. [111] prepared a French bread dough by fortifying it with 20% wheat bran of coarse (1.8 mm) and fine (18 µm) particle sizes and noted many differences in bread’s properties, e.g., loss of dough stability and increase of its viscosity, bread density and porosity during proofing, firmer crumb and stiffer crust as well as a decrease in mixer’s mechanical energy. Boita et al. [112] noted that the increased incorporation of wheat bran in the flour (from 25% to 100%) increased water absorption. On the other hand, it decreased stability, dough development time, extensibility, and viscosity. These results were attributed to the thinned and weakened gluten network formed by incorporating wheat bran in the dough. In order to reduce the negative impact of bran on the functional properties of the dough and the increased bioavailability of the minerals, Sanz Penella et al. [113] combined the addition of wheat bran with phytase and α-amylase.

Cereal processing by-products have also been used for the development of extruded snacks. For instance, Nascimento et al. [114] combined BSG (up to 30% in the dough) with rice flour in order to prepare extruded snacks with desirable characteristics compared to those prepared only with rice flour. Stojceska et al. [115] used a similar percentage (30%) of BSG to develop extruded ready-to-eat snacks with increased dietary fiber content (at least by 10%). On the other hand, the incorporation of wheat bran in extruded food products faces similar problems with bread application in spite of sensory characteristics and texture, e.g., by increasing the amount of bran in the mixture, the hardness and density of the products grew, while the crispness and the expansion volume are decreased [116,117]. It is thus vital to optimize the amount of added wheat bran. 

Cereals bran has also been suggested to fortify spaghetti. However, their increased incorporation is known to affect the cooking quality of pasta negatively, e.g., higher cooking losses and decreased water absorption [118]. Aravind et al. [119] prepared spaghetti from durum semolina substituted with different amounts (10–30%) of wheat bran and referred that the increased inclusion of bran led to higher cooking losses, decreased firmness, and greater surface roughness. On the other hand, the dietary fiber and antioxidants content was increased compared to the control pasta sample, while starch digestibility was not affected. On the other hand, Padalino et al. [120] investigated gluten-free spaghetti development using oat bran-rich (22%) in β-glucans and maize flour. According to their findings, the addition of the noted hydrocolloids improved the firmness and elasticity of the pasta, leading to low bulkiness and adhesiveness.

Finally, other cereal processing by-products such as rice distilling lees have been proposed as a potential substrate for the production of seasonings and flavors or to fortify cookies [121]. Ertaş [122] applied different processing techniques such as autoclave, microwave, and oven stabilization to improve the nutritional content of cookies. According to the results, the fortification with 30% microwave-treated bran provided cookies with high mineral content, while the autoclave-treated cookies showed the highest loss of phytic acid. The best sensory properties (e.g., appearance, flavor, color, and taste) were obtained for the cookies fortified with 10% of oven-treated bran.

## 7. Conclusions

The conventional utilization of cereal processing by-products for livestock feed and composting is a low added-value solution for the cereal sector that seeks more sustainable solutions within the stressing post-pandemic era and bioeconomy frame of our times. On the other hand, these by-products have considerable potential to be used as substrates for the production of different products for biotechnology, food, and pharmaceutical applications. The most sustainable strategy for the development of such supply chains is the biorefinery concept that is ideal for the valorization of side streams and the integration of recovery processes. Most of the research and market implementation studies conducted in the field deal with the valorization of wheat bran by-products and DDGS. After the recovery of valuable compounds such as proteins and arabinoxylans, a further valorization of the residual materials for the production of biofuels and other biobased products could increase the energy and material efficiency as well as the profit obtained from the downstream processing of the cereal processing by-products. To this line, innovative recovery approaches, non-thermal technologies, and more integral strategies should be further investigated together with tailor-made applications in foods and other sectors. Several compounds extracted from cereals, bran, and other waste streams have been referred to possess beneficial health claims for consumers, but more in vivo, human clinical trials regarding their digestibility, intake, and absorption in the body should be performed to validate these claims. The applications of cereal processing by-products or the corresponding recovered high added-value compounds in foods should also comply with the safety and quality regulations for human consumption, and thereby more efforts are needed in this direction. The most popular application is the fortification of bakery and other food products with cereals bran that brings not only health benefits and improved functional properties, but also problems in bread’s quality and organoleptic character. Therefore, more insights and more in-depth investigations are needed to address these issues prior to the commercialization of respective applications.

## Figures and Tables

**Table 1:** Processing steps during the dry and wet milling of different cereals.

Processes	Dry Milling				Wet Milling	
	Corn	Paddy Rice	Wheat	Oat	Corn	Barley
Cleaning	Yes	Yes			Yes	
Steeping					Yes	Yes
Conditioning	Yes		Yes			
Evaporation					Yes	
Germination						Yes
Processing/Breaking		Yes	Yes			
Coarse grinding					Yes, germ separation	
Degermination	Yes, germ separation					
Drying/Dehusking	Yes	Yes, husk separation, delivering brown rice				Yes, root separation, delivering malt
Aspiration	Yes					
Grading	Yes					
Sieving			Yes, twice	Yes		
Polishing		Yes, wastewater removal				
Oil extraction		Yes				
Destoning				Yes		
Grinding	Yes	Yes, delivering white rice	Yes, delivering flour	Yes, bran separation, delivering flour	Yes, bran separation	
Starch washing					Yes, starch separation	

**Table 2:** Functional components of cereal processing by-products and health properties (remade from [5]).

Cereal	By-Product	Target Functional Compounds	Health Properties	Examples
Rice	Bran, husk	Vitamins, proteins, dietary fiber, oil	Vitamins possess antioxidant activities, proteins show hypoallergenic properties, fiber prevents cardiovascular diseases	Rice bran contains 179–389 mg tocopherols and tocotrienols (Vitamin E compounds)/kg [5]
Corn	Bran, germ	Oil, insoluble dietary fiber	Oil reduces cholesterol levels, fiber prevents cardiovascular diseases	Corn kernels, bran and fiber contain 98–113 mg, 10.4–15.3 mg and 38–84 mg of ferulate phytosterol esters/kg, respectively [23]
Sorghum and millet	Bran	Phenolic compounds, phytosterols and policosanons	Phenolic compounds possess antioxidant properties, phytosterols and policosanons reduce cholesterol levels	Due to the high policosanol content, sorghum dry distiller grain hexane extracts significantly reduced cholesterol absorption by up to 17% and non-HDL plasma cholesterol by up to 70% in animal models [24]
Oat	Bran, oat mill waste	Soluble dietary fiber, β-glucan	Dietary fiber contributes to an increase in fecal bulk, β-glucan has been shown to reduce blood cholesterol	Oat bran contains at least 5.5% of β-glucan per dry mater and a total dietary fiber content of 16.0% dry matter [5]
Wheat	Bran, germ	Fiber, arabinoxylans	Bran fiber contributes to an acceleration of intestinal transit, and an increase in fecal bulk. Arabinoxylans contribute to a reduction of glucose level in the blood	Arabinoxylans are accounting for 10.9–26.0% of dry matter of bran. Health benefits of arabinoxylans are attributed to their prebiotic effects for obesity and other metabolic malfunctions, and ability to lower blood cholesterol and the post-prandial glycemic response [25]
Rye	Bran	Fiber, arabinoxylans, phytosterols	Dietary fiber contributes to normal bowel function. Arabinoxylans contribute to a reduction of glucose level in the blood	Phytosterol content in rye is 700–100 µg/g [26]. Daily doses of 1–3 g of plant sterols can reduce blood cholesterol in humans [5]
Barley	Spent grains	Dietary fiber, β-glucan	β-glucan contributes to the reduction of the blood glucose rise after meal, dietary fiber reduces cholesterol levels	Total phytosterols in barley oils (0.18–1.44 g/15 g oil) are able to significantly lower low-density lipoprotein (LDL) cholesterol at reasonable dosages of 15 mL/d (1 tablespoon/d) [27]

**Table 3:** Different approaches and technologies for the recovery of valuable compounds from different cereal processing by-products.

Cereal Processing By-Product	Target Compound	Recovery Method	Results	References
Oat mill waste	β-Glucan	Polysulfone membrane was applied in the pilot cross-flow module for the ultrafiltration of β-glucan containing feeds (<600 mg/L) recovered from the industrial oat mill waste	Two thirds (~67%) of β-glucan had been recovered	[32]
Rice by-products	Peptides	Combined treatment with proteases and sequential fractionation with one microfiltration (0.2 μm) and three polyethersulfone ultrafiltration (8, 5, and 4 kDa, respectively) steps	The retentate with the highest peptide content was the one recovered with the 8 kDa membrane	[60]
Wheat bran	Proteins	The adherent endosperm was removed from wheat bran with brushing before being extracted using the alkaline treatment and isoelectric precipitation	High nitrogen dispensability with minimum and maximum protein solubility pH values of 5.5 and 11.5, respectively.	[61]
Barley spent grain	Proteins	Coupling ultrasonic-assisted extraction with ultrafiltration	Recovery yield of 146 mg/g under the optimum conditions (solid-to-liquid ratio of 2 g/100 mL, intensity of 88.2 W/100 mL of extractant, and application of a 30-kDa membrane	[62]
Βrewers’ spent grains (BSG)	Arabinoxylans	Sequential fractionation using KOH of increasing strength (0.5, 1.0, and 4.0 M) of growing strength and 50 mM Na_2_CO_3_	Extraction of arabinoxylans with a lower ratio of arabinose/xylose as a factor of alkali’s power.	[63]
Rice bran	Proteins	Coupling alkaline extraction with microwave-assisted extraction	The optimum conditions were found to be a power of 1000 W, a water solid-to-liquid ratio of 0.89 g bran/10 mL, and an extraction time of 90 s. Microwaves enhanced by 1.5 fold the protein yield of alkaline extraction.	[64]

## Data Availability

Not applicable.
